# Dietary risk factors for non-communicable diseases in Kenya: findings of the STEPS survey, 2015

**DOI:** 10.1186/s12889-018-6060-y

**Published:** 2018-11-07

**Authors:** Valerian Mwenda, Martin Mwangi, Loise Nyanjau, Muthoni Gichu, Catherine Kyobutungi, Joseph Kibachio

**Affiliations:** 1 Field Epidemiology and Laboratory Training Programme, Ministry of Health, Nairobi, Kenya; 2 Non-communicable disease division, Ministry of Health, Nairobi, Kenya; 3 African Population and Health Research Center, Nairobi, Kenya; 4 The Institute of Global Health, Faculty of Medicine, University of Geneva (UNIGE), Geneva, Switzerland

**Keywords:** NCD, STEPS, Survey, Dietary

## Abstract

**Background:**

Burden of non-communicable diseases (NCD) is increasing worldwide. Risk factor surveillance informs public health interventions in NCD control. This study describes the dietary risk factors for NCD found in the Kenya STEPS survey, 2015.

**Methods:**

We performed secondary analysis of the STEPS dataset to determine prevalence of dietary NCD risk factors and their determinants. New variables were created; high dietary salt, defined as addition of salt while eating or intake of processed foods high in salt and high dietary sugar, defined as intake of processed foods or drinks high in sugar in most meals or addition of sugar to beverages already with sugar, on a daily basis. We used the World Health Organization definition of minimum required intake of fruits and vegetables as consumption of less than five servings of fruits and vegetables per day. Perceptions of respondents on diet and health were also assessed. Accounting for complex survey sampling, we calculated prevalence of the various dietary modifiable determinants and adjusted odds ratios (AOR) to identify factors independently associated with dietary NCD risk factors.

**Results:**

Of the 4484 individuals surveyed; mean age was 40.5 years (39.9–41.1 years), 60% were female. Prevalence of high reported dietary salt intake was 18.3% (95% CI 17.2%, 19.5%) and sugar 13.7% (95% CI 11.7–15.8%). Awareness of health risk from dietary salt was 88% and 91% for dietary sugar. Approximately 56% of the respondents were implementing strategies to reduce dietary salt and 54% were doing the same for dietary sugar. Only 6.0% (95% CI 4.3–7.6%) of the respondents reported intake of a minimum of five servings of both fruits and vegetables daily. Unhealthy diet was associated with being male (AOR 1.33, 95% CI 1.04, 1.70,), age below 46 years (AOR 1.78, 95% CI 1.42, 2.12) and being a student (AOR 15.6, 95% CI 2.44, 99.39).

**Conclusion:**

Dietary risk communication should be targeted to males and people under 45 years of age, especially students. Further research is necessary to understand the knowledge: practice mismatch on unhealthy diets.

## Background

Non-communicable diseases (NCDs) are illnesses usually of chronic nature, with slow progression, lengthy course and generally non-transmittable from one person to another [1]. They are the leading causes of morbidity and mortality worldwide currently, with projections of even more burden by 2030 [2]. According to World Health Organization (WHO) recent estimates, NCDs kill approximately 38 million people worldwide annually, with the four major contributors of this burden being cardiovascular diseases, cancers, respiratory diseases and diabetes, accounting for around 82% of all NCD deaths globally [3]. Low and middle income countries (LMICs) constitute the biggest proportion of this disease burden, with almost three quarters of global NCD deaths, 82% of all NCD premature deaths (below age of 70 years), and having the highest number of healthy years lost/disability adjusted life years worldwide [3–6]. It is projected that this imbalance of disease burden will continue tilting negatively in favour of low income settings such that by 2030 they will have eight times more deaths than the developed world [7]. This is despite the fact that NCDs currently receive minimal resource allocation from both governments and donor assistance relative to infectious disease [8]. In addition, most LMICs are still grappling with communicable diseases and injuries arising from violence, workplace accidents and road traffic crashes hence constituting the triple burden of disease [9]. These dangers, which themselves thrive in poverty and weak health systems, have a huge potential of retarding and finally crippling the economic development of the LMICs, creating a vicious cycle of poverty and ill-health [10].

In Kenya, the scenario replicates that in other developing nations; 50% of all adult hospital admissions and 55% of all mortalities are from NCD, with the leading causes being cardiovascular diseases and cancer [11]. There is a possibility of underestimation due to uncertain or unavailable mortality and morbidity data on NCD in Kenya. Though the Kenyan Ministry of Health has adopted the global vision of halting and reversing the global NCD threat, the country’s capacity to do so, especially in the context of the sustainable development goals (SDGs) is doubtful [12]. The Ministry of Health launched the Kenya National strategy for the prevention and control of NCD, 2015 to 2020, to guide the implementation of specific measures to address the burden [13]. The control of NCD requires careful consideration of the underlying factors and calls for holistic view of health using the bio-psycho-social model, and launching of preventive measures at all levels. However, since the current investments in NCD control are unlikely to bear fruits in the short-term, there is need for setting realistic goals for national programmes, and probably intensifying preventive measures and behavior change among adolescents and young adults by changing their physical, social and economic environments [4].

The WHO has identified surveillance of risk factors as a main pillar in the fight against NCDs by focusing on primary prevention through comprehensive, population-wide programmes targeting the major risk factors and their control [14]. With the main focus being the big four contributors of morbidity and mortality (cardiovascular diseases, cancers, chronic respiratory diseases and diabetes), the Global Action Plan 2013–2020 envisions reduction in mortality from NCD and sets targets for reduction of the main risk factors including harmful alcohol use, tobacco use, physical inactivity and unhealthy diet [15]. The STEPwise approach to risk factor surveillance (STEPS) was designed by WHO to enable countries to collect core data on major risk factors that drive the disease burden, with a flexible structure to allow different countries to adapt it to their individual situations [16]. It involves three steps: a questionnaire-based assessment of socio-economic, nutritional and behavioural information; step two involves simple physical measurements and step three involves biochemical measurements of blood sugar and cholesterol. It employs a food questionnaire to estimate levels of dietary intake, especially salt, sugar, fruits and vegetables; this is recognized as one of the approaches in population-based settings [17]. Kenya carried out its first STEPS survey for NCD risk factors in 2015, with main objective being to determine the prevalence of behavioural and biological risk factors as well as that of oral health and unintentional injuries. This was the first nationally representative survey to explore the burden of NCD risk factors in the Kenyan population. The main objective of the secondary analysis of data from the STEPS survey, Kenya, 2015 was to determine the prevalence, awareness and perception, as well as the determinants of dietary NCD risk factors.

## Methods

### Overview of STEPS Kenya, 2015 methods

The STEPS Kenya 2015 survey was a cross-sectional household survey targeting adults between the ages of 18 to 69 years. The sample size was determined to be 6000 to allow for national estimates as per sex and residence (rural or urban). A total of 4500 individuals were successfully interviewed.

The STEPS survey was carried out from April to June 2015. Its focus was the four main behavioural risk factors of NCDs (tobacco use, harmful use of alcohol, unhealthy diets and physical inactivity), the four key biological risk factors for NCDs (overweight and obesity, raised blood pressure, raised blood lipids and raised blood glucose) as well as burden of unintentional injuries and oral health. It was a national, cross-sectional survey that used the fifth national sample surveys and evaluation programme (NASSEP V) sampling frame from the Kenya National Bureau of Statistics, developed using the enumeration areas generated from the 2009 Kenya Population and Housing Census. A three stage cluster sample design was used; in the first stage, 200 clusters (100 urban and 100 rural) were selected. In the second stage, a uniform sample of 30 households were selected from the listed households in each cluster, while in the third stage, one individual was randomly selected from all eligible listed household members. Socio-demographic and behavioral information was collected in step 1. A full description of the survey methods is available elsewhere [18]. The survey questionnaire was adapted from the WHO STEPS instrument, and included a section for dietary history on salt, sugar, fat, fruits and vegetable intakes. The dietary questionnaire relied on reported intakes: the respondents were given examples of foods high in salt and sugar. For salt, these included foods that have been altered from their natural state, such as njugu-karanga (fried groundnuts), packaged salty snacks, canned salty food including pickles and preserves, salty food prepared at a fast food restaurant, cheese, bacon and processed meat. For sugar, the examples given were soda (carbonated drinks) like fanta, coca cola,7-up, Afya,

Softa, Vimto, biscuits, wafers, cakes, candy, sweets and chocolate and alike. For fruits and vegetables intake, a nutrition card was used to show examples of local fruits and vegetables, with each picture representing the size of a serving (one serving).

### New variables

We derived three new variables from the dietary part of the survey dataset on salt, sugar, fruits and vegetables intake. These included reported high dietary salt, reported high dietary sugar, and reported low intake of fruits and vegetables. Reported high dietary salt intake was defined as addition of salt at the table while eating or intake of processed foods high in salt on a daily basis. Reported high dietary sugar was defined as addition of sugar to drinks already served with sugar or intake of processed foods or drinks high in sugar on a daily basis. The information for the two new variables on sugar and salt intake was derived from the responses to the series of questions asking about amounts taken and foods high in salt or sugar. Inadequate intake of fruits and vegetables was defined as consumption of less than five servings of fruits and vegetables per day, as recommended by the WHO [19]. During the survey, intake of fruits and vegetables was gauged by asking questions on the frequency in terms of days and servings. A composite variable; unhealthy diet, made up of the three variables (high dietary salt, high dietary sugar and low intake of fruits and vegetables) when present together was also defined. All these new outcome variables created were then compared across the various independent variables used in this study including sex, age, marital status, level of education, occupation, residence and socioeconomic status (wealth band). These new variables are not a standard part of the STEPS survey, but were deemed important at the secondary data analysis for further informing policy and planning. Finally, we determined the proportions of the respondents who were aware of the harms of unhealthy diet, those regarding intake of healthy diet as very important and those actively taking measures to reduce these risks at the time of the survey. This was used to determine how the respondents perceived unhealthy diet as a major risk factor for NCD.

### Statistical analysis

Statistical analysis was performed using STATA version 14.1, StataCorp LLC, USA. We used the “svy” method in STATA to create estimates that adjust for the complex, multi-level sampling design, including stratifying sampling by Kenyan regions and enumerator areas. We computed adjusted odds ratios for each exposure variable while controlling for all the other variables (confounders) in the model with 95% confidence intervals that excluded the null (AOR equal to 1.0) considered statistically significant. Our analysis included 4484 respondents after omitting records that had missing values for the independent and dependent variables from the initial survey sample size of 4500 respondents.

## Results

Out of the 4500 individuals who were successfully interviewed, 4484 were included in this analysis. Of these, 60% were female, 42% had secondary education and above while 13% had no formal education **(**Table 1**).** The mean age was 40.5 years (39.9–41.1 years). Sixty-six percent of the respondents were married, 40% were unemployed while 62% were rural residents.

**Table 1:** Socio-demographic characteristics of the respondents, Kenya STEPS survey, 2015

Characteristic	% proportions (95% CI)	Sample size (*N* = 4484)	
		Unweighted, *n*	Weighted, *n*
Age groups			
18–29	46.0 (43.6, 48.4)	1484	2062
30–39	23.3 (21.6, 25.1)	1253	1045
40–49	15.5 (14.1, 17.0)	793	695
50–59	9.9 (8.8, 11.0)	541	443
60–69	5.3 (4.7, 6.1)	413	239
Marital status			
Not married	23.2 (21, 25.5)	783	1039
Married	65.5 (63.1, 67.8)	3045	2938
Formerly married	11.3 (10, 12.7)	656	507
Residence			
Rural	61.9 (59.4, 64.4)	2404	2776
Urban	38.1 (35.6, 40.6)	2080	1708
Education level			
No formal education	12.6 (11.4, 13.8)	754	563
Primary education	45.6 (43.3, 47.9)	2086	2043
Secondary and above	41.9 (39.5, 44.3)	1644	1877
Wealth band			
Poorest	18.9 (17.4, 20.5)	898	848
Second	20.9 (19.3, 22.6)	897	937
Middle	18.3 (16.8, 19.8)	897	818
Fourth	18.6 (16.7, 20.6)	896	832
Richest	23.4 (21.0, 25.9)	896	1049
Occupation			
Unemployed	40.1 (37.9, 42.4)	1873	1799
Employed	59.9 (57.6, 62.1)	2611	2685

### Prevalence of NCD risk factors

The prevalence of various dietary risk factors were as follows: reported high dietary salt 18.3% (95% CI 17.2–19.5%), reported high dietary sugar 13.7% (11.7–15.8%), and fruits and vegetable servings less than five per day 94.0% (95% CI 92.4–95.7%). A breakdown of the individual dietary risk factors is shown in Table 2.

**Table 2:** Prevalence of various individual dietary risk factors, STEPS survey, 2015

Variable	Age-group	Prevalence (%, 95% CI)		
		Male	Female	Overall
Always add salt to food before eating/while eating	18–29	28.8 (22.1–35.5)	18.9 (13.8–24.1)	23.6 (18.5–28.8)
	30–44	25.1 (19.5–30.6)	23.2 (17.7–28.6)	24.1 (19.7–28.5)
	45–59	21.9 (15.9–27.9)	18.2 (13.4–23.0)	20.1 (16.1–24.1)
	60–69	24.7 (16.1–33.3)	20.7 (10.8–30.7)	22.6 (15.3–30.0)
	18–69	26.2 (21.5–31.0)	20.3 (15.9–24.6)	23.2 (19.1–27.2)
Often consume processed foods high in salt	18–29	7.0 (3.8–10.1)	4.2 (2.3–6.1)	5.5 (3.9–7.2)
	30–44	3.7 (2.1–5.3)	3.8 (1.1–6.6)	3.8 (2.2–5.3)
	45–59	3.3 (1.2–5.3)	2.8 (0.0–5.8)	3.0 (1.4–4.7)
	60–69	1.6 (0.0–4.2)	0.0 (0.0–0.0)	0.8 (0.0–2.0)
	18–69	5.0 (3.2–6.8)	3.7 (2.1–5.2)	4.3 (3.2–5.5)
Consumes processed foods high in sugar	18–29	2.1 (0.6–3.5)	2.9 (0.7–5.2)	2.5 (1.2–3.9)
	30–44	1.9 (0.5–3.4)	0.3 (0.1–0.5)	1.1 (0.4–1.9)
	45–59	1.1 (0.0–2.3)	0.0 (0.0–0.1)	0.6 (0.0–1.2)
	60–69	0.0 (0.0–0.0)	0.0 (0.0–0.0)	0.0 (0.0–0.0)
	18–69	1.8 (0.7–2.8)	1.5 (0.4–2.6)	1.6 (0.8–2.4)
Always adds sugar to beverages before intake	18–29	31.6 (25.3–38.0)	24.4 (17.6–31.2)	27.9 (21.9–33.8)
	30–44	27.9 (20.6–35.2)	26.9 (20.7–33.0)	27.4 (21.6–33.2)
	45–59	30.0 (19.4–40.6)	26.6 (20.4–32.8)	28.3 (21.7–34.9)
	60–69	23.4 (15.7–31.0)	30.7 (21.0–40.5)	27.1 (20.0–34.3)
	18–69	29.7 (24.2–35.2)	25.9 (20.4–31.4)	27.7 (22.7–32.8)
Intake of less than 5 servings of fruits and/or vegetables daily	18–29	93.1 (90.0–96.2)	95.6 (93.9–97.3)	94.4 (92.4–96.5)
	30–44	92.9 (90.2–95.6)	94.9 (92.9–96.9)	93.9 (92.0–95.8)
	45–59	95.6 (93.3–97.9)	93.2 (90.2–96.1)	94.4 (92.5–96.3)
	60–69	89.4 (83.2–95.7)	91.4 (87.1–95.8)	90.5 (86.7–94.2)
	18–69	93.2 (91.1–95.3)	94.8 (93.3–96.3)	94.0 (92.4–95.7)

### Factors associated with unhealthy diet

High dietary salt intake was associated with male gender (AOR 1.53, 95% CI 1.33–1.76), urban residence (AOR 1.46 (95% CI 1.24–1.72) and being employed (AOR 1.56, 95% CI 1.26–1.93), student (AOR 1.94, 95% CI 1.39–2.71) and homemaker (AOR 1.73, 95% CI 1.35–2.22) compared with those unemployed. High dietary sugar intake was associated with urban residence (AOR 1.4, 95% CI 1.10–1.78), having attained secondary education and above or at least 12 years of formal schooling (AOR 2.15, 95% CI 1.59, 2.90) and being a student (AOR 6.66, 95% CI 2.59–17.16). Males had higher odds of low fruits and vegetables intake **(**Table 3**)**. Unhealthy diet (high salt and sugar, low fruits and vegetables) was associated with being male (AOR 1.33, 95% CI 1.04–1.70), age below 46 years (AOR 1.78, 95% CI 1.42–2.12) and being a student (AOR 15.6, 95% CI 2.44–99.39) **(**Table 4).

**Table 3:** Determinants of the four nutritional risk factors: high reported dietary salt and sugar and low intake of fruits and vegetables, STEPS survey Kenya, 2015

	High dietary salt (Adjusted OR, 95% CI)	High dietary sugar (Adjusted OR, 95% CI)	Low fruit intake (Adjusted OR, 95% CI)	Low vegetable intake (Adjudged OR, 95% CI)
Age group				
18–29	1.0	1.0	1.0	1.0
30–39	0.83 (0.70, 0.98)	1.10 (0.87, 1.40)	1.03 (0.83, 1.27)	0.78 (0.54, 1.13)
40–49	0.68 (0.56, 0.83)	0.79 (0.62, 1.02)	1.05 (0.82, 1.33)	0.54 (0.34, 0.87)
50–59	0.53 (0.42, 0.67)	0.77 (0.58, 1.03)	1.07 (0.81, 1.41)	0.32 (0.19, 0.55)
60–69	0.46 (0.33, 0.62)	0.54 (0.38, 0.76)	1.15 (0.81, 1.62)	0.23 (0.12, 0.45)
Sex				
Female	1.0	1.0	1.0	1.0
Male	1.53 (1.33, 1.76)	1.02 (0.84, 1.24)	1.41 (1.17, 1.69)	1.80 (1.25, 2.60)
Residence				
Rural	1.0	1.0	1.0	1.0
Urban	1.46 (1.24, 1.72)	1.4 (1.1, 1.78)	0.90 (0.72, 1.12)	1.12 (0.7, 1.78)
Education level				
No formal education	1.0	1.0	1.0	1.0
Primary complete	1.06 (0.85, 1.32)	2.09 (1.61, 2.7)	0.28 (0.22, 0.35)	0.07 (0.05, 0.10)
Secondary and above	1.21 (0.95, 1.55)	2.15 (1.59, 2.9)	0.16 (0.12, 0.21)	0.05 (0.03, 0.09)
Marital status				
Not married	1.0	1.0	1.0	1.0
Married	0.83 (0.69, 1.00)	0.73 (0.55, 0.96)	1.01 (0.79, 1.28)	0.74 (0.47, 1.18)
Wealth band				
Poorest	1.0	1.0	1.0	1.0
Second	1.14 (0.93, 1.41)	0.84 (0.64, 1.09)	0.69 (0.56, 0.86)	0.24 (0.15, 0.37)
Middle	1.05 (0.84, 1.31)	1.13 (0.84, 1.52)	0.39 (0.31, 0.51)	0.25 (0.15, 0.41)
Fourth	1.75 (1.38, 2.21)	1.25 (0.90, 1.72)	0.34 (0.26, 0.45)	0.23 (0.13, 0.41)
Richest	1.64 (1.26, 2.14)	1.07 (0.74, 1.56)	0.24 (0.17, 0.35	0.10 (0.05, 0.22)
Occupation				
Not employed	1.0	1.0	1.0	1.0
Employed	1.56 (1.26, 1.93)	0.84 (0.64, 1.10)	0.76 (0.60, 0.97)	1.58 (0.96, 2.60)
Student	1.94 (1.39, 2.71)	6.66 (2.59, 17.16)	0.79 (0.52, 1.20)	2.66 (1.24, 5.70)
Homemaker	1.73 (1.35, 2.22)	1.26 (0.92, 1.74)	1.01 (0.77, 1.33)	2.98 (1.75, 5.05)

**Table 4:** Determinants of overall unhealthy dietary practices (high salt, high sugar, low fruits and vegetables when present together), STEPS survey Kenya, 2015

Unhealthy diet	Adjusted odds ratio
	AOR (95% CI)
Age group (years)	
46–69	1.00
18–45	1.78 (1.42, 2.12)
Sex	
Female	1.00
Male	1.33 (1.04, 1.70)
Residence	
Rural	1.00
Urban	1.19 (0.89, 1.59)
Education level	
High school incomplete	1.00
High school complete	1.29 (0.97, 1.71)
Marital Status	
Single	1.00
Married	0.94 (0.73, 1.21)
Wealth band (divided into quintiles)	
Below middle	1.00
Middle	1.07 (0.78, 1.47)
Above middle	0.92 (0.67, 1.27)
Occupation	
Unemployed	1.00
Employed	0.84 (0.58, 1.21)
Student	15.57 (2.44, 99.39)
Homemaker	1.25 (0.82, 1.93)

### Awareness and perception on sugar and salt intake as NCD risk factors

**Fig. 1:**
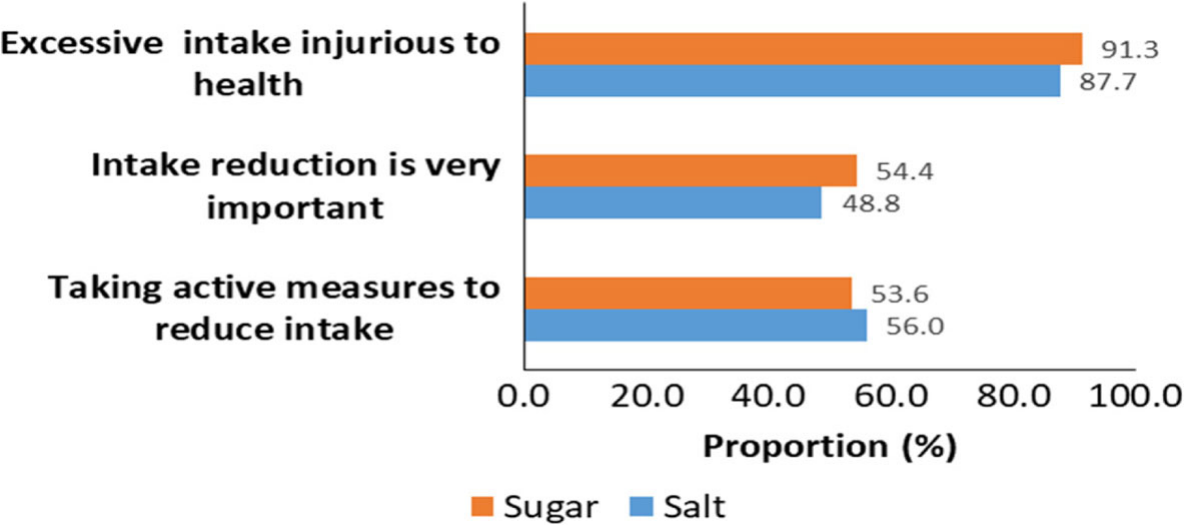
Awareness and perceptions on unhealthy consumption of salt and sugar, Kenya STEPS survey, 2015

Though awareness of the health dangers of high dietary salt and sugar intake was reported by majority of the respondents (87.7% and 91.3% respectively), only about half of the respondents regarded reduction of the same in diet as very important, and an approximately equal proportion was implementing strategies to reduce dietary sugar and salt intake **(**Fig. 1).

## Discussion

This analysis showed that NCD dietary risk factors are prevalent in Kenya, and are differentiated by age, sex, and occupation. We also noted an awareness: perception/practice mismatch regarding dietary sugar and salt as NCD risk factors. The survey respondents were generally middle-aged and majority were females, most likely due to the fact that this was a household based survey and women are the most consistent inhabitants of the home in the Kenyan context.

Approximately one fifth of the respondents reported high dietary salt intake. This is comparable with findings from a meta-analysis of population-based studies on association between salt intake and hypertension in rural and urban communities in low and middle income countries in 2014 that found a prevalence of high salt intake of 21% to 90% [20], as well as a meta analysis of other studies from the continent on salt intake, done in 2016 [21]. Discretionary salt intake (salt addition at the table) is a major contributor to high salt intake globally; however it is difficult to control in a wide scale through legislation [22]. The contribution of salt intake from processed foods is relatively less in LMICs but could be on the rise due to urbanization and changes in lifestyles, with urban residents frequently preferring processed fast foods. Salt intake control strategies generally are two pronged; reduction of salt in processed foods through legislation to govern the food industry as well as enforcement of existing laws, and secondly, creating consumer awareness and public education, especially to combat discretionary excessive salt intake [23–25]. There is evidence of reduction in NCD prevalence through cost-effective salt intake reduction strategies in Sub-Saharan Africa, in studies done in Nigeria [26] and Ghana [24], with hypertension as the NCD end-point target under study. Lower salt intake has been associated with lower blood pressure, as well as reduced prevalence of stroke and fatal coronary heart disease in adults; these combined are the leading contributors to the current NCD burden globally [27–30]. Reduction of salt intake at population level has not been shown to negatively impact iodine status in settings where iodized salt is applied as a strategy to reduce iodine deficiency [31]. The biggest contributor to high dietary salt intake from our study is addition of salt at the table before or during eating; this can be mitigated by sustained public education efforts on the dangers of high dietary salt intake.

High dietary sugar intake was reported by one in every seven respondents; a study in South Africa reported doubling of intake of sweetened beverages over a five-year period, from 29 to 60% [32]. However, for beverages prepared at home, our study only focused on ‘topping up’ of already sugared beverages, and this could partly explain the difference in the two studies. High dietary sugar intake is associated with several health hazards including insulin resistance and subsequently type 2 diabetes, abnormal lipids, hypertension, obesity and several other cardio-metabolic risk factors [33–35]. The observed mismatch between awareness and practice on health hazards of high dietary salt and sugar intake has been observed in other nutritional surveys in different settings, including studies by Grimes et al. in Australia [36], Nasreddine et al. in Lebanon [37] and Magalhães et al. in Angola [38]. We hypothesize that one of the explanations for this mismatch is that majority in the population lack knowledge on measures to reduce dietary sugar and salt intake, even though they are aware of the health hazards.

Only 6% of the survey respondents were consuming the minimum daily recommended five servings of fruits and vegetables. Low fruit and vegetable intakes have also been noted in other STEPS surveys whose findings have been reported to the WHO [39]. Several determinants of fruits and vegetable intake in various communities globally have been suggested, including ethnicity, cultural differences, preferences, availability and affordability [40, 41]. Overall, there is an upward trend in fruits and vegetables intake globally [42], and there are particular initiatives in Africa to promote this trend, including supporting urban fruits and vegetables farming and large-scale commercial farming of the same in rural areas [43]. There is evidence that adequate intake of fruits and vegetables is associated with reduction in all-cause mortality, especially from cardiovascular causes [44].

Association between unhealthy diet with male gender and younger age has been noted in other studies, [20, 23, 38]. This is likely due to increase in rural-urban migration for career opportunities among the youth and adoption of diets high in processed foods from restaurants and fast food as a consequence of rapid urbanization. The most prevalent component of unhealthy diet was low intake of recommend minimum levels of fruits and vegetables; additional studies to determine knowledge an attitudes on their intake is warranted since they are readily available in most Kenyan settings.

A major limitation of this study was the fact that the questionnaire relied on food recall and reported dietary intakes, hence discerning the accuracy of the reports was difficult.

## Conclusion

Dietary NCD risk factors are prevalent in the Kenyan adult population, and most of the assessed variables fell short of the WHO recommended dietary intakes for attainment of the NCD control targets [45]. The information generated in the survey can be used in policy formulation, designing intervention strategies, while evaluation of the remedial measures can be achieved by comparison with future STEPS surveys nationally and internationally.

## Abbreviations

NASSEP V: fifth national sample surveys and evaluation programme
NCD: Non-communicable disease
SDGs: Sustainable development goals
STEPS: Stepwise approach to surveillance
WHO: World Health Organization
